# Weighted Schatten *p*-Norm Low Rank Error Constraint for Image Denoising

**DOI:** 10.3390/e23020158

**Published:** 2021-01-27

**Authors:** Jiucheng Xu, Yihao Cheng, Yuanyuan Ma

**Affiliations:** 1 College of Computer and Information Engineering, Henan Normal University, Xinxiang 453007, China; xjc@htu.edu.cn (J.X.); c_yihao@126.com (Y.C.); 2 Engineering Technology Research Center for Computing Intelligence and Data Mining, Xinxiang 453007, China

**Keywords:** image denoising, low rank representation, weighted schatten *p*-norm, low rank error constraint

## Abstract

Traditional image denoising algorithms obtain prior information from noisy images that are directly based on low rank matrix restoration, which pays little attention to the nonlocal self-similarity errors between clear images and noisy images. This paper proposes a new image denoising algorithm based on low rank matrix restoration in order to solve this problem. The proposed algorithm introduces the non-local self-similarity error between the clear image and noisy image into the weighted Schatten *p*-norm minimization model using the non-local self-similarity of the image. In addition, the low rank error is constrained by using Schatten *p*-norm to obtain a better low rank matrix in order to improve the performance of the image denoising algorithm. The results demonstrate that, on the classic data set, when comparing with block matching 3D filtering (BM3D), weighted nuclear norm minimization (WNNM), weighted Schatten *p*-norm minimization (WSNM), and FFDNet, the proposed algorithm achieves a higher peak signal-to-noise ratio, better denoising effect, and visual effects with improved robustness and generalization.

## 1. Introduction

Image contains a lot of information. However, owing to the noise, important information may lost in the process of image acquisition, compression, transmission, and storage, which brings inconvenience to the subsequent image processing. Therefore, image denoising is necessary in image preprocessing [1]. The degradation model for the denoising problem can be expressed as: Y=X+N, where *N* is usually assumed to be additive white Gaussian noise with a standard deviation of $\sigma_{n}$. The purpose of image denoising is to restore a clean image *X* from the noise observation *Y* as accurately as possible while maintaining important detailed features (such as edges and textures). Image denoising is a typical ill-posed problem in mathematics, which can be solved using prior knowledge of an image [2]. In the past few decades, many effective image prior knowledge models have been developed, such as regularization methods that are based on total variation [3,4,5], sparse representation [6,7], low rank representation [8,9], nonlocal self-similarity [10,11], and deep learning [12], et al.

Recently, the image prior method based on nonlocal self-similarity [13,14] and low rank matrix approximating [15,16,17,18] can better preserve image edge details while denoising, which has achieved some success in image denoising [19,20]. Low rank matrix approximation aims to recover the underlying low rank matrix from degraded observations. It is widely used in computer vision and machine learning. Low rank matrix approximation can be divided into two categories: low rank matrix decomposition and rank minimization. This study focuses on the rank minimization method; its main idea is to reconstruct the data matrix by imposing additional rank constraints on the estimated matrix [21]. Cai et al. proposed the nuclear norm minimization (NNM) model and applied it to image denoising [16], while NNM tends to excessively reduce the singular values and treat different singular values equally. In practical problems, the larger singular value of the data matrix quantifies the information of its basic principal direction. In the image data matrix, the larger singular value provides the main edge and texture information. Therefore, the shrinking of the larger singular value should be reduced, and the smaller singular value should be shrunk more, in order to restore the image from the damaged image. Obviously, the traditional NNM model is not flexible enough to deal with such problems and it cannot accurately estimate the rank of the matrix. To solve this problem, Nie et al. proposed the Schatten *p*-norm model, which obtains better estimation results for matrix rank than NNM [22]. However, similar to the standard nuclear norm, most of the models that are based on Schatten *p*-norm treat all singular values equally, and cannot estimate the rank of matrix commendably in many practical problems (such as image inverse problem). Gu et al. proposed a weighted nuclear norm minimization (WNNM) model to further improve the flexibility of the NNM model [23]. When compared with NNM, WNNM assigns different weights to different singular values, which makes the value of soft threshold more reasonable. Then, Xie et al. proposed a more flexible model, the weighted Schatten *p*-norm minimization (WSNM) model, which assigns weights to different singular values and better approximates the original low rank matrix approximation problem. Among them, WNNM is a special case of WSNM [24]. However, WSNM has a high time complexity. Zhang et al. proposed a modified Schatten *p*-norm minimization (MSpNM) model to reduce the total number of iterations, thereby reducing the time complexity of calculation, in order to reduce the time complexity [25]. However, the model is difficult to learn accurate prior knowledge when the image is seriously damaged by noise. Zha et al. proposed a rank residual constraint (RRC) model that could progressively approximate the underlying low rank matrix via minimizing the rank residual and achieve a better estimate of the desired image [19].

In order to solve the above problems, this paper studies the weighted Schatten *p*-norm minimization model, and attempts to integrate the nonlocal self- similarity errors of clear and noisy images into the weighted Schatten *p*-norm minimization denoising algorithm. The image denoising problem is converted to the problem of minimizing Schatten *p*-norm and low rank error constraints, and then finds the optimal low rank matrix. Secondly, the generalized soft threshold algorithm [26] is applied to solve the optimal solution of the low rank matrix in the weighted Schatten *p*-norm minimization and the optimal solution in the low rank error constraint, and further obtain a more robust low rank matrix optimal solution that is based on the mean of the two optimal solutions. Finally, an image denoising algorithm that is based on the weighted Schatten *p*-norm low rank error constraint (WSNLEC) is proposed, and the standard image Data set Set12 is used for simulation experiments to verify the effectiveness of the proposed algorithm.

## 2. Related Work

The proposed algorithm is based on the minimization of the weighted Schatten *p*-norm and the nonlocal self-similarity of the image. The weighted Schatten *p*-norm perform low rank regularization effectively, and image nonlocal similarity can preserve the edge details commendably in image denoising process.

### 2.1. Weighted Schatten p-Norm Minimization

When the number of columns or rows of a matrix is much greater than the rank of the matrix, it is said that the matrix has low rank. The low rank property of a matrix can also be described as the existence of a small number of non-zero eigenvalues after singular value decomposition. The rank minimization method reconstructs the data matrix by adding additional rank constraints to the estimated matrix. The main idea is to give a matrix *Y* and obtain a low rank matrix *X*, then rank minimization is defined as:

(1) $$\hat{X}=\arg\min_{X}{∥Y-X∥}_{F}^{2}+\lambda R\left(X\right),$$

where ${∥Y-X∥}_{F}^{2}$ is the data fidelity term, ${∥∥}_{F}$ represents the *F*-norm, λR(X) denotes the low rank regularization term, and λ is a parameter that is used to balance the loss function and the low rank regularization term.

Because the direct rank minimization is NP-difficult and ill-posed, this problem is generally solved by alternatively minimizing the nuclear norm of the estimated matrix. However, nuclear norm minimization tends to excessively reduce the rank component and treat different rank components equally, which limits its ability and flexibility. In order to effectively carry out low rank regularization, paper [24] proposed a weighted Schatten *p*-norm minimization model, where the weighted Schatten *p*-norm of the matrix $X\in {ℜ}^{b\times m}$ is expressed as:

(2) $${∥X∥}_{w,S_{p}}^{p}=\sum_{i=1}^{\min\{b,m\}}w_{i}{\sigma_{i}}^{p}=tr\left(W\Delta^{p}\right),$$

where 0<p≤1, $\sigma_{i}$ denotes the *i*-th singular value of matrix *X*, $w=[w_{1},\ldots ,w_{\min\{b,m\}}]$, $w_{i}\geq 0$ represents the non-negative weight assigned to $\sigma_{i}$, $w_{i}$, and $\sigma_{i}$ are the diagonal elements of the diagonal matrix *W* and Δ, respectively.

Given a matrix *Y*, the nonconvex weighted Schatten *p*-norm minimization model aims to find a matrix *X*, which is as close to *Y* as possible under the conditions of *F*-norm data fidelity and weighted Schatten *p*-norm regularization:

(3) $$\hat{X}=\arg\min_{X}{∥Y-X∥}_{F}^{2}+{∥X∥}_{w,S_{p}}^{p}.$$

### 2.2. NonLocal Self-Similarity

The main idea of image prior method that is based on low rank representation is that the data matrix formed by nonlocal similar patches in natural images has low rank property. Among them, nonlocal self-similarity characterizes the repeatability of texture and structure reflected by natural image in nonlocal area, which is, for an image patch $x_{i}$, a large number of image patches that are similar to the image patch can be found in the image, and these similar image patches are called similar patches [13].

This study is based on the nonlocal self-similarity of images. The clear image *X* with size *N* is divided into *n* overlapping image patches, i=1,2,…,n. For each image patch $x_{i}$, use the block matching algorithm that was proposed in [27] to search for the *m* image patches that are most similar to the image patch $x_{i}$ to form a matrix $X_{i}$, namely $X_{i}=\left\{x_{i,1},x_{i,2},\ldots ,x_{i,m}\right\}$. Because all of the image patches have similar structure in each data matrix, the constructed data matrix $X_{i}$ has low rank property.

The corresponding low rank matrix $X_{Ci}$ is obtained from each similar group $X_{i}$, and the optimal solution of $X_{Ci}$ is obtained by the Schatten *p*-norm, which can be expressed as:

(4) $${\hat{X}}_{Ci}=\arg\min_{X_{Ci}}{∥X_{i}-X_{Ci}∥}_{F}^{2}+{∥X_{Ci}∥}_{w,S_{p}}^{p}.$$

The similarity group $Y_{i}$ in the noise image is similar to the clear image, namely $Y_{i}=\left\{y_{i,1},y_{i,2},\ldots ,y_{i,m}\right\}$, where $y_{i,m}$ represents the *m*-th similar patch of the *i*-th similar group $Y_{i}$. The problem of image denoising can be transformed into recovering potential clear image *X* from noisy image *Y* using low rank representation and solving the optimal solution *X* of low rank matrix in noisy image, which can be expressed as:

(5) $${\hat{X}}_{i}=\arg\min_{X_{i}}{∥Y_{i}-X_{i}∥}_{F}^{2}+{∥X_{i}∥}_{w,S_{p}}^{p}.$$

The nonlocal self-similarity method can preserve the edge details in the image denoising process.

## 3. Principle and Method of WSNLEC

The WSNLEC algorithm that is proposed in this paper merges the low rank error constraints into the weighted Schatten *p*-norm minimization denoising algorithm, and the image denoising problem is transformed into minimizing the Schatten *p*-norm and low rank error constraints, and then the optimal low rank matrix problem is obtained.

### 3.1. Low Rank Error

It is difficult to estimate an accurate low rank matrix from the image *Y*, due to the influence of noise. Specifically, there is an error between the low rank matrix $X_{C}$ of the original clear image *X* obtained from the Equation (4) and the estimated low rank matrix *X* obtained from the Equation (5). The error *R* can be expressed as:

(6) $$R=X-X_{C}.$$

It is necessary to enhance the accuracy of the low rank matrix, which is to make the error sufficiently small, in order to improve the performance of image denoising. Therefore, this paper introduces the low rank error into the weighted Schatten *p*-norm minimization denoising model, and Equation (5) can be improved as:

(7) $${\hat{X}}_{i}=\arg\min_{X_{i}}{∥Y_{i}-X_{i}∥}_{F}^{2}+{∥X_{i}-X_{Ci}∥}_{w,S_{p}}^{p}.$$

We use the Schatten *p*-norm to regularize the low rank error, and then obtain the optimal low-rank matrix by minimizing the low-rank error. The accuracy of low rank matrix increases with the decrease of low rank error.

### 3.2. Core Idea of WSNLEC

The clear image *X* is unknown in the process of image denoising. Therefore, it is difficult to obtain the real low rank matrix, but it can be approximately expressed by the accurate estimation of the low rank matrix. For the algorithm shown in this paper, the FFDNet method first proposed in [28] is used to preprocess the noise image *Y* to obtain the image $Y_{D}$, and then initialization $Y_{D}$ in order to obtain more accurate estimation value of the low rank matrix $X_{D}$.

This paper fuses the low rank error constraints into the weighted Schatten *p*-norm minimization denoising algorithm, and the image denoising problem is converted to minimizing the Schatten *p*-norm and low rank error constraints, and then obtain the optimal low rank matrix problem, in order to improve the performance of the image denoising algorithm. The minimization that is based on the weighted Schatten *p*-norm error constraint can be expressed as:

(8) $$\begin{array}{c} {\hat{X}}_{i}=\arg\min_{X_{i}}{∥Y_{i}-X_{i}∥}_{F}^{2}+{∥X_{i}∥}_{w,S_{p}}^{p}+{∥X_{i}-X_{Ci}∥}_{w,S_{p}}^{p}. \end{array}$$

Under the assumption of low rank, we could use the low rank matrix approximation method to obtain estimation matrix $X_{i}$ that can be obtained from $Y_{i}$, according to the degeneration model of additive white Gaussian noise. Subsequently, we apply the proposed weighted Schatten *p*-norm error constraint model to the estimation of $X_{i}$, and the corresponding optimization problem can be defined as:

(9) $$\begin{array}{c} {\hat{X}}_{i}=\arg\min_{X_{i}}\frac{1}{\sigma_{n}^{2}}{∥Y_{i}-X_{i}∥}_{F}^{2}+{∥X_{i}∥}_{w,S_{p}}^{p}+{∥X_{i}-X_{Ci}∥}_{w,S_{p}}^{p}, \end{array}$$

where $\sigma_{n}^{2}$ is the noise variance, ${∥Y_{i}-X_{i}∥}_{F}^{2}$ denotes the *F*-norm fidelity term, ${∥X_{i}∥}_{w,S_{p}}^{p}$ represents the low rank regularization, and ${∥X_{i}-X_{Ci}∥}_{w,S_{p}}^{p}$ is the low rank error constraint term.

Equation (9) is divided into two sub-problems, one is to solve the low rank matrix in the weighted Schatten *p*-norm minimization problem and the other is to solve the low rank matrix in the low rank error constraint problem. Finally, we use the mean solving method to obtain the final low rank matrix, and Equation (9) can be rewritten as:

(10) $$\begin{array}{c} {\hat{X}}_{i}=\arg\min_{X_{i}}\frac{1}{\sigma_{n}^{2}}{∥Y_{i}-X_{i}∥}_{F}^{2}+\frac{1}{2}\left({∥X_{i}∥}_{w,S_{p}}^{p}+{∥X_{i}-X_{Ci}∥}_{w,S_{p}}^{p}\right). \end{array}$$

### 3.3. Solution Method

In this paper, we use the generalized soft-thresholding algorithm (GST) to solve the proposed algorithm [26]. Given *p* and $w_{i}$, there is a specific threshold:

(11) $$\tau_{p}^{GST}\left(w_{i}\right)={\left(2w_{i}\left(1-p\right)\right)}^{\frac{1}{2-p}}+w_{i}p{\left(2w_{i}\left(1-p\right)\right)}^{\frac{p-1}{2-p}},$$

where, if $\sigma_{i}<\tau_{p}^{GST}\left(w_{i}\right)$, then $\delta_{i}=0$ is the global minimum; otherwise, the best value will be obtained at a non-zero point. For any $\sigma_{i}\in \left(\tau_{p}^{GST}\left(w_{i}\right),+\infty \right)$, $f_{i}\left(\delta \right)$ has a unique minimum value $S_{p}^{GST}\left(\sigma_{i};w_{i}\right)$, which can be obtained by solving the following equation:

(12) $$S_{p}^{GST}\left(\sigma_{i};w_{i}\right)-\sigma_{i}+w_{i}p{\left(S_{p}^{GST}\left(\sigma_{i};w_{i}\right)\right)}^{p-1}=0.$$

Generally, the larger *j*-th singular value of *X* is of greater importance than the smaller singular value $\sigma_{j}\left(X_{i}\right)$. Because the larger singular value of the matrix provides the information of its basic principal direction, and the larger singular value in the image matrix provides the main edge and texture information. Therefore, the shrinking of the larger singular value should be reduced, and the smaller singular value should be shrunk more, in order to recover the clear image from the damaged image. Similarly, the *j*-th singular value of the optimal solution of Equation (9) has the same attribute, and then the larger the value of $\delta_{j}\left({\hat{X}}_{i}\right)$, the smaller the value that should be reduced. Therefore, an intuitive way to set the weight is that the weight should be inversely proportional to $\delta_{j}\left({\hat{X}}_{i}\right)$ [20]:

(13) wj=cncn(δj11pp(δj11pp(X^i)+ε),

where *n* is the number of similar patches in $Y_{i}$, ε sets to $10^{-16}$ to avoid division by zero, and $c=2\sqrt{2}\sigma_{n}^{2}$. Because $\delta_{j}\left({\hat{X}}_{i}\right)$ is not available before estimating $\hat{X}$, it can be initialized as:

(14) $$\delta_{j}\left({\hat{X}}_{i}\right)=\sqrt{\max\{\sigma_{j}^{2}\left(Y_{i}\right)-n\sigma_{n}^{2},0\}}.$$

We use the iterative regularization scheme that was adopted in [12], this scheme adds the filtered residual back to the denoised image, as shown below:

(15) $$Y^{\left(k\right)}={\hat{X}}^{\left(k-1\right)}+\alpha \left(Y-{\hat{X}}^{\left(k-1\right)}\right),$$

where *k* represents the number of iterations and α is a relaxation parameter.

Finally, all of the denoised image patches are merged to form the denoised image ${\hat{X}}^{k}$.

### 3.4. WSNLEC in Image Denoising

In this paper, we use the weighted Schatten *p*-norm as the regularization term to ensure the low rank of the key information in the image. There is a certain error between the low rank matrix solved from the noisy image and the real low rank matrix due to the impact of the noise in the image. Therefore, WSNLEC introduces a low rank error constraint, and it proposes a gray image denoising algorithm that is based on the weighted Schatten *p*-norm low rank error constraint. The low rank error constraint reduces the error between the obtained low rank matrix and the real low rank matrix. Hence, a more accurate low rank matrix optimal solution is obtained and the denoising performance of the algorithm is improved.

Algorithm 1 summarizes the image denoising algorithm that is based on the weighted Schatten *p*-norm low rank error constraint.
**Algorithm 1:** Weighted Schatten *p*-norm low rank error constraint (WSNLEC) for Image Denoising.**Input:** Noisy image *Y*(1) Initialize ${\hat{X}}^{0}=Y$, $Y_{D}$;(2) **For** *k* = 1 : *K* **do**(3)    Iterative regularization $Y^{\left(k\right)}={\hat{X}}^{\left(k-1\right)}+\alpha \left(Y-{\hat{X}}^{\left(k-1\right)}\right)$;(4)    Construct similar groups ${Y_{i}}^{k}$ and ${Y_{Di}}^{k}$ by block matching method [27];(5)    **For** each local image patch $y_{i}$ **do**(6)       Estimate the *k*-th weight vector ${W_{j}}^{k}$ by wj=cncn(δj11pp(δj11pp(X^i)+ε);(7)       Update $X_{i}^{k}$ by GST algorithm;(8)       Update $X_{Ri}^{k}$ by GST algorithm;(9)       Update ${\hat{X}}_{i}^{k}$ by Equation (10);(10)   **End For**(11)   Aggregate ${\hat{X}}_{i}^{k}$ to form the denoised image ${\hat{X}}^{k}$;(10) **End For****Output:** Denoised image ${\hat{X}}^{k}$

## 4. Experimental Results and Analysis

In order to test the performance of the proposed WSNLEC in image denoising, we compare it with four representative algorithms: block matching three-dimensional (3D) filtering (BM3D) [27], weighted nuclear norm minimization (WNNM) [23], weighted Schatten *p*-norm minimization method (WSNM) [24], and FFDNet [28]. Subsequently, analyze the experimental results.

### 4.1. Experimental Setup

**Figure 1:**
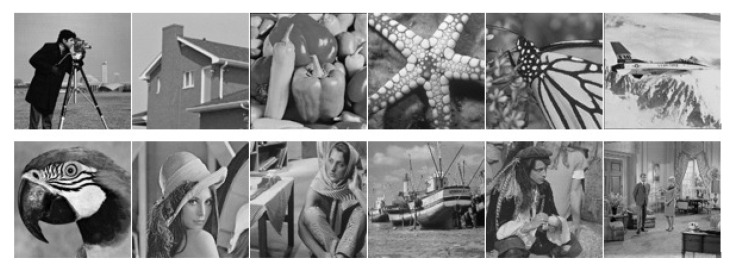
Set12 test image set.

WSNLEC needs to set several parameters. The parameter settings are the same as WSNM in order to ensure the validity and reliability of the experiment. The power *p* value ranges from 0.05 to 1, and the step size is 0.05. Finally, p={1.0,0.85,0.75,0.7,0.1,0.05} and the corresponding noise level is set as $\sigma_{n}=\{20,30,50,60,75,100\}$. In order to test the effectiveness of the algorithm, the public dataset Set12 is used in the experiment (as shown in Figure 1). All of the experiments in this dataset are implemented using MATLAB R2016a on Windows 10 with an Intel Core i5-3470 CPU at 3.20 GHz and 8.0 GB memory.

In order to obtain the noise image, add Gaussian white noise to the test image, and the noise standard deviations σ are 20, 30, 50, 60, 75, and 100, respectively. The size of overlapped image patches is different at various noise level. When the noise standard deviation σ≤20, the size of overlap patch is 6×6; when the noise standard deviation 20≤σ≤40, the size of overlap patch is 7×7; when the noise standard deviation 40≤σ≤60, the size of overlap patch is 8×8; when the noise standard deviation σ>60, the size of overlap patch is 9×9.

### 4.2. Experimental Results of Noise Reduction Algorithms with Different Standard Deviations

We use the peak signal-to-noise ratio (PSNR) as the evaluation criterion. The higher the PSNR value, the better the image denoising effect. Table 1, Table 2, Table 3, Table 4, Table 5 and Table 6 show the PNSR values under different standard deviations between the proposed algorithm and other comparison algorithms.

Table 1 shows the standard deviation σ = 20 of Gaussian noise. Table 2 shows the standard deviation σ = 30 of Gaussian noise. Table 3 shows the standard deviation σ = 50 of Gaussian noise. Table 4 shows the standard deviation σ = 60 of Gaussian noise. Table 5 shows the standard deviation σ = 75 of Gaussian noise. Table 6 shows the standard deviation σ = 100 of Gaussian noise. The highest values of PNSR shown in Table 1, Table 2, Table 3, Table 4, Table 5 and Table 6 are expressed in bold. The PSNR value of each denoising algorithm decreases with the increase of noise standard deviation, as shown from Table 1, Table 2, Table 3, Table 4, Table 5 and Table 6. The PSNR values of WSNLEC are higher than other comparison algorithms at almost all noise levels, and the average PSNR value is higher than other comparison algorithms.

Among them, the results of the experiment are compared with the results of the FFDNet algorithm used in preprocessing in order to prove the validity of the experiment. The results prove that the PNSR value of the algorithm that is proposed in this paper is higher than the FFDNet algorithm under different standard deviations. That is, the performance of the WSNLEC algorithm is better than the FFDNet algorithm.

In the case that the noise standard deviation σ = 20, the PSNR value of WSNLEC is 0.72 dB, 2.09 dB, 0.37 dB, and 0.23 dB, which are higher than BM3D, WNNM, WSNM, and FFDNet, respectively. When the noise standard deviation σ = 30, the PSNR value of WSNLEC is 0.76 dB, 1.80 dB, 0.37 dB, and 0.21 dB higher than BM3D, WNNM, WSNM, and FFDNet, respectively. When the noise standard deviation σ = 50, the PSNR value of WSNLEC is 0.87 dB, 1.39 dB, 0.49 dB, and 0.23 dB higher than BM3D, WNNM, WSNM, and FFDNet, respectively. When the noise standard deviation σ = 60, the PSNR value of WSNLEC is 0.88 dB, 1.44 dB, 0.55 dB, and 0.27 dB higher than BM3D, WNNM, WSNM, and FFDNet, respectively. When the noise standard deviation σ = 75, the PSNR value of WSNLEC is 0.89 dB, 1.61 dB, 0.51 dB, and 0.22 dB higher than BM3D, WNNM, WSNM, and FFDNet, respectively. When the noise standard deviation σ = 100, the PSNR value of WSNLEC is 1.36 dB, 1.51 dB, 0.5 dB, and 0.21 dB higher than BM3D, WNNM, WSNM, and FFDNet, respectively.

While using the nonlocal self-similarity of the image, the proposed algorithm adds a low rank error constraint that is based on the weighted Schatten p-norm and reduces the error between the estimated low rank matrix and real low rank matrix. The experimental results show that the proposed algorithm has better denoising performance, effectiveness and feasibility.

### 4.3. Experimental Results of Denoising Algorithms for Different Test Images

**Figure 2:**
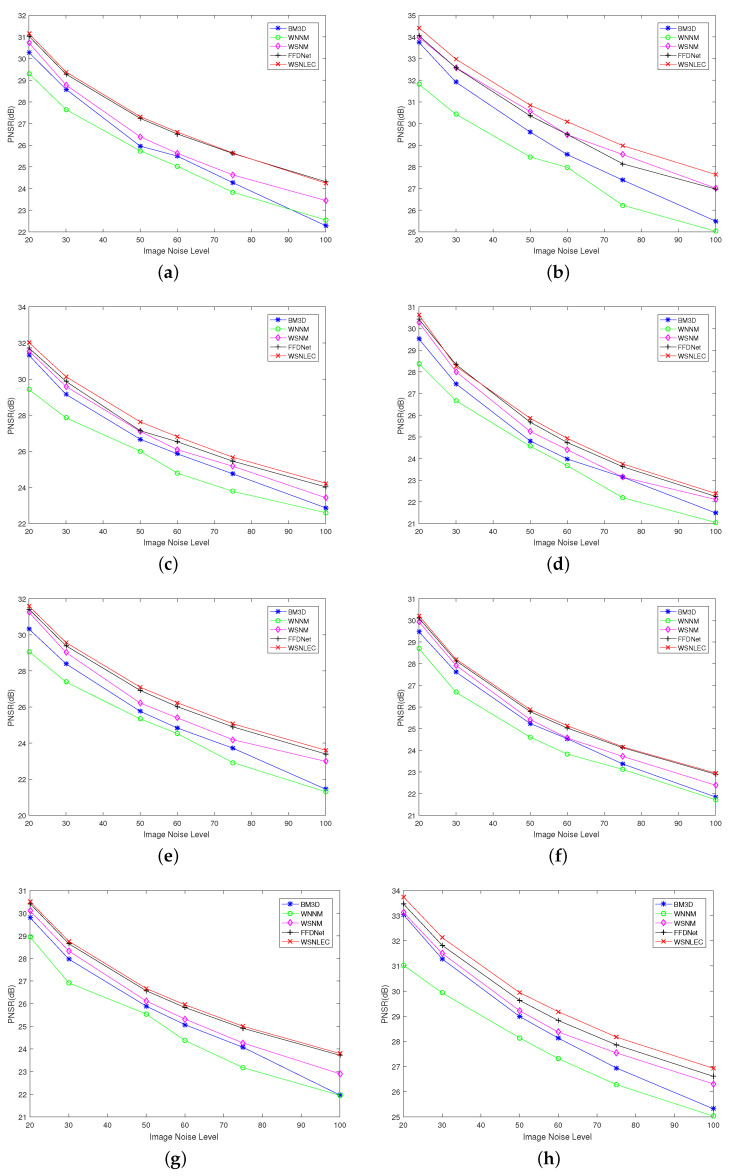

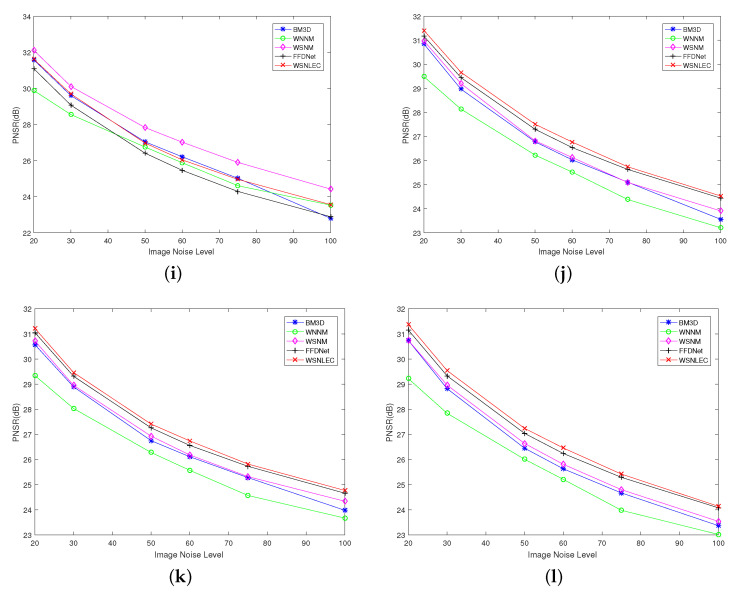
PNSR values of all algorithms under different noise standard deviations (*σ*) for each image in the Set12 image data set. (**a**) C.Man. (**b**) House. (**c**) Peppers. (**d**) Starfish. (**e**) Monarch. (**f**) Airplane. (**g**) Parrot. (**h**) Lena. (**i**) Barbara. (**j**) Boat. (**k**) Man. (**l**) Couple.

For each image in the Set12 image dataset, under different standard deviations, the PNSR values of the proposed algorithm and all of the comparison algorithms are represented by line graph in order to demonstrate the advantages of the proposed algorithm clearly, as shown in Figure 2.

Figure 2 shows the PNSR values of all the algorithms under different noise standard deviations (σ=20,30,50,60,75,100) for each image. The test images are C. Man, House, Peppers, Starfish, Monarch, Airplane, Parrot, Lena, Barbara, Boat, Man, and Couple. The red line represents the algorithm proposed in this paper. The blue line represents BM3D. The green line represents WNNM. The purple line represents WSNM, and the gray line represents FFDNet. It is clear that, except for Barbara image, whose PNSR value of WSNM is the highest, in all other test images, the PNSR value of WSNLEC is significantly higher than that of other comparison algorithms.The results show that, in most test images, the performance of the proposed algorithm is better than other comparison algorithms.

Among them, the results of the experiment are compared with the results of the FFDNet algorithm used in preprocessing in order to prove the validity of the experiment. The results prove that the PNSR value of the algorithm that is proposed in this paper is higher than the FFDNet algorithm under different standard deviations. It can be seen that the performance of the algorithm that is proposed in this paper is better than the pre-processed FFDNet algorithm.

### 4.4. Visual Effects of Different Denoising Algorithms

**Figure 3:**
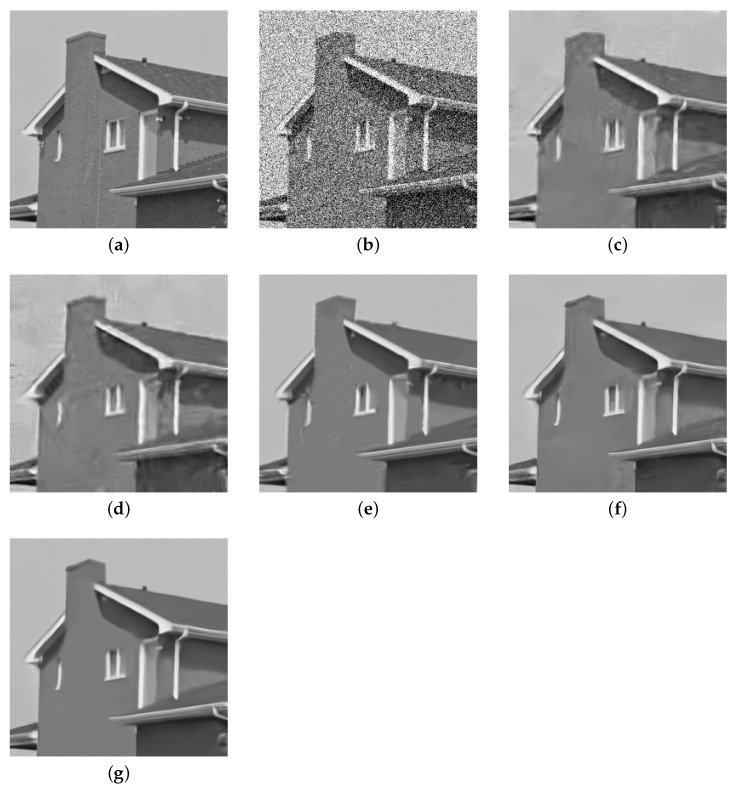
Denoising results on image “House” with noise level *σ* = 50. (**a**) is the clear image; (**b**) is the noisy image (*σ* = 50); (**c**) is the denoised image of BM3D (PNSR = 28.57); (**d**) is the denoised image of WNNM (PNSR = 28.46); (**e**) is the denoised image of WSNM (PNSR = 30.56); (**f**) is the denoised image of FFDNet (PNSR = 29.50); and, (**g**) is the denoised image of WSNLEC (PNSR = 30.85).

We use the House test image with noise standard deviation σ = 50 for simulation experiment in order to show the visual effect of the denoising algorithm better. Figure 3 shows the test result of House. It is obvious that the wsnlec algorithm performs well in the denoising effect, the edge and detail information are better protected, especially the line contour, the background is smoother, and better visual experience is obtained. However, we have no great contribution in texture. Although our algorithm does not express all details clearly, it shows the best visual effect in the comparison algorithm effect.

**Figure 4:**
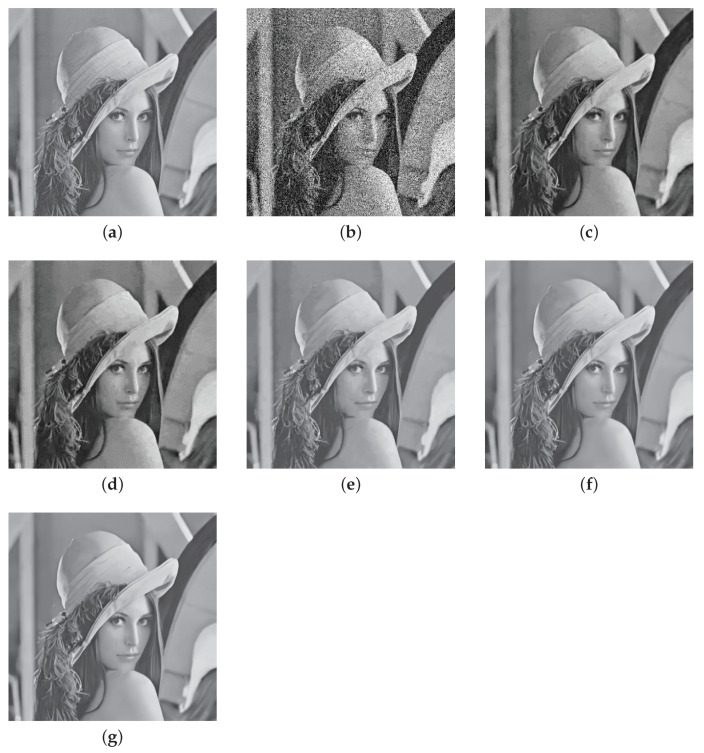
Denoising results on image “Lena” with noise level σ = 30. (**a**) is the clear image; (**b**) is the noisy image (σ = 30); (**c**) is the denoised image of BM3D (PNSR = 31.28); (**d**) is the denoised image of WNNM (PNSR = 29.93); (**e**) is the denoised image of WSNM (PNSR = 31.50); (**f**) is the denoised image of FFDNet (PNSR = 31.82); (**g**) is the denoising image of WSNLEC (PNSR = 32.13).

Furthermore, we use the Lena test image with noise standard deviation σ = 30 for simulation experiment. Figure 4 shows the test result of Lena. It can be clearly seen that WSNLEC has a better denoising effect than other comparison algorithms. The facial texture is smoother, and the edge details of the facial features are clearer. Although there is no clear display of texture details, it shows the best visual effect in the contrast algorithm.

The proposed algorithm adds a low rank error constraint on the basis of the weighted Schatten *p*-norm, reduces the error between the estimated low rank matrix and real low rank matrix, and retains the image detail features well while removing the noise. From the above experimental results and analysis, it is obvious that WSNLEC not only has strong denoising performance and obtains a higher peak signal-to-noise ratio, but it also can produce visual effects better.

## 5. Conclusions

The Schatten *p*-norm optimization method is normally employed to obtain prior information from noisy images directly, and little effort is paid on the nonlocal self-similarity errors between clear images and noisy images. Aiming at these problems, a weighted Schatten *p*-norm low rank error constraint algorithm for image denoising is proposed, which introduces the nonlocal self-similarity error between the clear image and noise image to the weighted Schatten *p*-norm minimization model. The low rank error is constrained to obtain a better low rank matrix and improve the denoising performance of the algorithm. Firstly, the algorithm divides the problem of solving the optimal low rank matrix into two sub-problems. Subsequently, the generalized soft threshold algorithm is used to solve the low rank matrix in Schatten *p*-norm and low rank error constraint, respectively. Finally, the mean value of them is taken as the final low rank matrix. The proposed algorithm is compared with four classical and effective image denoising algorithms (BM3D, WNNM, WSNM, and FFDNet), and the experimental results show that the algorithm can robustly solve the low rank matrix, with higher PSNR, better denoising effect, and greater practicability and effectiveness. In the future, we will continue to optimize the algorithm, reduce the time complexity of the algorithm, and apply it to color images.

## Figures and Tables

**Table 1:** Denoising PNSR (dB) results of different denoising algorithms under standard deviations (σ = 20).

σ = 20					
**Image**	**BM3D**	**WNNM**	**WSNM**	**FFDNet**	**WSNLEC**
C.Man	30.28	29.31	30.72	31.03	**31.15**
House	33.75	31.81	33.99	34.06	**34.42**
Peppers	31.32	29.42	31.55	31.72	**32.02**
Starfish	29.52	28.38	30.28	30.43	**30.63**
Monarch	30.32	29.07	31.25	31.42	**31.59**
Airplane	29.47	28.70	29.93	30.10	**30.21**
Parrot	29.80	28.95	30.10	30.42	**30.51**
Lena	33.04	31.30	33.13	33.47	**33.75**
Barbara	31.58	29.88	**32.11**	31.09	31.62
Boat	30.85	29.49	30.98	31.17	**31.41**
Man	30.57	29.34	30.72	31.05	**31.22**
Couple	30.74	29.22	30.74	31.15	**31.37**
Average	30.94	29.57	31.29	31.43	**31.66**

**Table 2:** Denoising PNSR (dB) results of different denoising algorithms under standard deviations (σ = 30).

σ = 30					
**Image**	**BM3D**	**WNNM**	**WSNM**	**FFDNet**	**WSNLEC**
C.Man	28.58	27.65	28.78	29.28	**29.38**
House	31.91	30.43	32.58	32.57	**32.97**
Peppers	29.15	27.85	29.59	29.87	**30.13**
Starfish	27.45	26.67	28.01	**28.34**	28.25
Monarch	28.39	27.39	29.02	29.39	**29.57**
Airplane	27.61	26.68	27.92	28.13	**28.21**
Parrot	27.97	26.92	28.33	28.65	**28.76**
Lena	31.28	29.93	31.50	31.82	**32.13**
Barbara	29.60	28.55	**30.31**	29.07	29.69
Boat	28.97	28.14	29.20	29.45	**29.67**
Man	28.89	28.03	28.95	29.31	**29.46**
Couple	28.82	27.84	28.96	29.33	**29.54**
Average	29.05	28.01	29.43	29.60	**29.81**

**Table 3:** Denoising PNSR (dB) results of different denoising algorithms under standard deviations (σ = 50).

σ = 50					
**Image**	**BM3D**	**WNNM**	**WSNM**	**FFDNet**	**WSNLEC**
C.Man	25.96	25.74	26.39	27.24	**27.32**
House	29.61	28.46	30.56	30.36	**30.85**
Peppers	26.67	26.01	27.10	27.41	**27.63**
Starfish	24.81	24.57	25.25	25.68	**25.86**
Monarch	25.76	25.34	26.22	26.92	**27.10**
Airplane	25.24	24.61	25.42	25.79	**25.88**
Parrot	25.89	25.54	26.12	26.57	**26.67**
Lena	28.99	28.14	29.22	29.63	**29.94**
Barbara	27.04	26.75	**27.83**	26.41	26.97
Boat	26.78	26.23	26.82	27.30	**27.51**
Man	26.75	26.28	26.93	27.26	**27.41**
Couple	26.45	26.01	26.64	27.04	**27.24**
Average	26.66	26.14	27.04	27.30	**27.53**

**Table 4:** Denoising PNSR (dB) results of different denoising algorithms under standard deviations (σ = 60).

σ = 60					
**Image**	**BM3D**	**WNNM**	**WSNM**	**FFDNet**	**WSNLEC**
C.Man	25.50	25.03	25.63	26.52	**26.61**
House	28.57	27.98	29.49	29.50	**30.09**
Peppers	25.86	24.79	26.09	26.53	**26.81**
Starfish	23.98	23.67	24.41	24.74	**24.93**
Monarch	24.84	24.53	25.40	26.02	**26.24**
Airplane	24.54	23.83	24.57	25.03	**25.14**
Parrot	25.07	24.37	25.32	25.83	**25.95**
Lena	28.13	27.31	28.37	28.83	**29.18**
Barbara	26.21	25.88	**27.02**	25.46	26.04
Boat	26.03	25.52	26.13	26.54	**26.77**
Man	26.11	25.57	26.18	26.56	**26.74**
Couple	25.63	25.21	25.81	26.24	**26.46**
Average	25.87	25.31	26.20	26.48	**26.75**

**Table 5:** Denoising PNSR (dB) results of different denoising algorithms under standard deviations (σ = 75).

σ = 75					
**Image**	**BM3D**	**WNNM**	**WSNM**	**FFDNet**	**WSNLEC**
C.Man	24.27	23.83	24.63	25.62	**25.64**
House	27.39	26.23	28.57	28.14	**28.98**
Peppers	24.75	23.78	25.17	25.45	**25.67**
Starfish	23.14	22.19	23.14	23.62	**23.75**
Monarch	23.72	22.92	24.19	24.90	**25.07**
Airplane	23.37	23.12	23.72	24.12	**24.16**
Parrot	24.07	23.17	24.26	24.91	**25.00**
Lena	26.94	26.28	27.54	27.86	**28.17**
Barbara	25.02	24.61	**25.90**	24.29	24.96
Boat	25.10	24.38	25.09	25.63	**25.74**
Man	25.28	24.57	25.32	25.73	**25.82**
Couple	24.67	23.98	24.80	25.29	**25.42**
Average	24.81	24.09	25.19	25.48	**25.70**

**Table 6:** Denoising PNSR (dB) results of different denoising algorithms under standard deviations (σ = 100).

σ = 100					
**Image**	**BM3D**	**WNNM**	**WSNM**	**FFDNet**	**WSNLEC**
C.Man	22.29	22.55	23.45	**24.30**	24.25
House	25.50	25.03	27.02	26.98	**27.65**
Peppers	22.87	22.60	23.43	24.03	**24.23**
Starfish	21.49	21.05	22.11	22.25	**22.39**
Monarch	21.45	21.31	22.99	23.40	**23.61**
Airplane	21.85	21.72	22.39	22.90	**22.95**
Parrot	21.96	21.95	22.90	23.72	**23.80**
Lena	25.32	25.03	26.31	26.61	**26.93**
Barbara	22.80	23.53	**24.42**	22.89	23.56
Boat	23.56	23.21	23.91	24.44	**24.53**
Man	23.98	23.67	24.34	24.66	**24.77**
Couple	23.37	23.02	23.54	24.08	**24.14**
Average	23.04	22.89	23.90	24.19	**24.40**

## Symbols Explanation

*Y*	Noisy image
*X*	Clear image
*N*	Noise
λ	Regularization parameter
${\hat{X}}^{k}$	Denoised image
$X_{i}$	Similarity group of clear image
$X_{Ci}$	Low rank matrix from clear image
$Y_{i}$	Similarity group of noisy image
*R*	Low rank matrix error
$Y_{D}$	Apply FFDNet method to preprocess the noise image *Y* and obtain the image $Y_{D}$
$\sigma_{n}^{2}$	Noise variance
*p*	Power
$w_{i}$	Weight
$\sigma_{i}$,$\delta_{i}$	The *i*-th singular value of matrix
$\tau_{p}^{GST}\left(w_{i}\right)$	Threshold
ε	Constant term
*k*	The number of iterations
α	Relaxation parameter
